# Transition to the new race/ethnicity data collection standards in the Department of Veterans Affairs

**DOI:** 10.1186/1478-7954-4-7

**Published:** 2006-07-06

**Authors:** Min-Woong Sohn, Huiyuan Zhang, Noreen Arnold, Kevin Stroupe, Brent C Taylor, Timothy J Wilt, Denise M Hynes

**Affiliations:** 1 Midwest Center for Health Services and Policy Research, Hines, IL, USA; 2 Feinberg School of Medicine, Northwestern University, Chicago, IL, USA; 3 VA Information Resource Center, Hines, IL, USA; 4 Minneapolis VA Center for Chronic Disease Outcomes Research, Minneapolis, MN, USA; 5 University of Minnesota School of Medicine, Minneapolis, MN, USA; 6 Loyola University Chicago, Maywood, IL, USA

## Abstract

**Background:**

Patient race in the Department of Veterans Affairs (VA) information system was previously recorded based on an administrative or clinical employee's observation. Since 2003, the VA started to collect self-reported race in compliance with a new federal guideline. We investigated the implications of this transition for using race/ethnicity data in multi-year trends in the VA and in other healthcare data systems that make the transition.

**Methods:**

All unique users of VA healthcare services with self-reported race/ethnicity data in 2004 were compared with their prior observer-recorded race/ethnicity data from 1997 – 2002 (N = 988,277).

**Results:**

In 2004, only about 39% of all VA healthcare users reported race/ethnicity values other than "unknown" or "declined." Females reported race/ethnicity at a lower rate than males (27% vs. 40%; p < 0.001). Over 95% of observer-recorded data agreed with self-reported data. Compared with the patient self-reported data, the observer-recorded White and African American races were accurate for 98% (kappa = 0.89) and 94% (kappa = 0.93) individuals, respectively. Accuracy of observer-recorded races was much worse for other minority groups with kappa coefficients ranging between 0.38 for American Indian or Alaskan Natives and 0.79 for Hispanic Whites. When observer-recorded race/ethnicity values were reclassified into non-African American groups, they agreed with the self-reported data for 98% of all individuals (kappa = 0.93).

**Conclusion:**

For overall VA healthcare users, the agreement between observer-recorded and self-reported race/ethnicity was excellent and observer-recorded and self-reported data can be used together for multi-year trends without creating serious bias. However, this study also showed that observation was not a reliable method of race/ethnicity data collection for non-African American minorities and racial disparity might be underestimated if observer-recorded data are used due to systematic patterns of inaccurate race/ethnicity assignments.

## Background

In 1997, the Office of Management and Budget (OMB) released the revised standards for the collection of race and ethnicity known as Statistical Directive 15 that federal agencies were mandated to comply by January, 2003 [1-3]. The most significant changes in the new standards included self-identification as the preferred data collection method and the ability to report multiple races for an individual. For researchers who use data from multiple years for disease surveillance or tabulation of utilization and cost trends by race groups, this transition involved two methodological issues. One was how to handle races for those who identify themselves with more than one race. Sometimes called the "bridging," this issue concerns how to assign multiracial persons to a single race category. The other was a more fundamental question of whether the prevalence of a particular disease, treatment, or outcomes would be comparable over time in race/ethnicity categories.

This study examines how this federal mandate affects the collection and use of race/ethnicity data for a large federal agency, the Department of Veterans Affairs (VA). Until 2003, the VA had collected data on race and ethnicity for all its healthcare users based on observation (e.g., registration clerk's or clinician's perception) [4]. In compliance with the OMB standards, the VA changed to self-identification as its preferred method of data collection. Now the underlying meaning of race has fundamentally changed (e.g., from appearance to self-perception) and the key question is whether the old data based on observation can be used together with the new, self-reported data.

The transition to the OMB standards is not an issue unique to the VA. Previous studies have examined the implications of this transition for state public health data [2,5], focusing mainly on "bridging" issues. The VA experience additionally involved issues related to change in data collection methods from observation to self-identification. Considering that many hospitals in the private sector still collect race data by observation [6] and have yet to make this transition for more standardized and reliable race/ethnicity data [7-9], this study can inform private-sector hospital administrators of the issues involved in making this transition.

The objective of this study is to examine the effect of this transition on the research use of race/ethnicity data for multi-year trends in the VA, specifically focusing on how comparable race data collected under two different methods are and what the effect of bridging may be on different race categories when one try to map multiracial race values to the old single races for the same individuals. Previous studies have examined data quality issues on race in the VA data [4,10,11], but none have examined the effect of this transition in the VA.

## Methods

### Study design and population

The Institutional Review Board at the Edward Hines, Jr. VA Hospital approved the study including a HIPAA waiver of authorization. In this study, we examined race data for all users of the Veterans Health Administration (VHA) healthcare services from fiscal years 1997 through 2004. A fiscal year in the VA runs from October 1^st ^of the previous year to September 31^st ^of the current year. All years henceforth are fiscal years unless otherwise noted.

The year 2004 is the first full year for which self-reported race/ethnicity is available within the VA healthcare data. For this reason, we used all VA healthcare users in 2004 as the baseline population, and identified all patients who had valid self-reported race values recorded in the VA healthcare utilization data in that year. A valid race value is defined in this study as one of the legitimate race/ethnicity categories excluding "unknown" or "declined."

They were then merged with healthcare utilization records in the years between 1997 and 2002 to capture all observer-recorded race values for the same individuals. 2003 was the transition year. For the first seven months of the year (until May 2003), race data were collected according to the old standards and for the rest of the year, according to the new standards. We did not use race data from 2003 due to concerns of data integrity during this transition year.

### Data sources

The VHA Medical SAS Inpatient (often called the Patient Treatment Files or PTF) and Outpatient (Outpatient Clinic Files or OPC) Datasets were used as main data sources [14-17]. These national patient-level datasets capture information collected at VHA healthcare sites and entered in the VHA electronic medical record system, including demographic data [18]. Before May 2003, the Inpatient Datasets recorded race values in one variable (RACE) and, after the implementation of the new standards, they captured race in six different variables (RACE1 – RACE6) to accommodate individuals who identify themselves with more than one race. The Outpatient Datasets contain similarly all records for outpatient care and the new race values were collected in seven new variables (RACE1 – RACE7) since 2004.

The new race variables encoded both the race and data collection method in one value (e.g., "AP" indicates proxy-reported Native Hawaiian or Other Pacific Islander). Three data collection methods could be recorded, including self-identification, observation, and proxy-reporting. In this study, a self-reported race value is one whose data collection method code explicitly indicated "self-identification." A multiracial individual was identified as such only when multiple self-identified race values were present on the same record for the individual (e.g., the same hospitalization or the same outpatient clinic visit). When different races were recorded on two or more records, we considered them to be inconsistent rather than multiracial.

Both Inpatient and Outpatient Datasets were combined to compile a list of all individuals who reported their race in the 2004 datasets. The same datasets for 1997 – 2002 were used to compile a list of individuals with valid observer-recorded race values.

When linking the data over time, we used social security numbers (SSNs), sex, and two parts of date of birth (e.g., year, month, or day) as linkage variables [19]. There were 8.4 million unique SSNs for all VHA users in years 1997 – 2002 and 2004. Of these, 1.7 percent (97,236) did not match according to these three criteria and were not used in the analysis.

### Statistical analysis

Self-reported race/ethnicity data for 2004 were compared with the observer-recorded data in the VA administrative files for 1997 – 2002 for the matching individuals. Since the OMB Statistical Directive 15 considers race/ethnicity a social construct and the self-reported race/ethnicity "accurate by definition" [3,5], we used the self-reported race/ethnicity as the gold standard to examine the accuracy of the old, observer-recorded data. For each old value, five different accuracy and agreement measures were computed. These measures included sensitivity, specificity, positive and negative predictive values, and kappa.

Sensitivity indicates the probability that the old race/ethnicity is correct according to the new race/ethnicity. Specificity indicates the probability that the old race/ethnicity correctly excludes a person who is not of that race/ethnicity. Positive predictive value indicates the probability that a person of a race/ethnicity according to the old data are actually of that race/ethnicity. Negative predictive value indicates the probability that a person not of a race/ethnicity in the old data are actually not of that race/ethnicity. Finally, we used Cohen's kappa as a measure of agreement between the old and new data [20]. According to Landis and Koch [21], a kappa coefficient larger than 0.8 indicates an excellent agreement.

To examine how the "bridging" issue may affect the VA race data, we used four bridging techniques that assign the whole person to a single race/ethnicity group using the smallest group, the largest group, or the largest group other than White, or assign equal fractions to all race groups reported. For example, an individual who identified oneself as White, African American, and Asian would be assigned to Asian using the smallest group assignment, White using the largest group assignment, and African American using the largest group other than White assignment methods. On the other hand, the same individual would be assigned equally to all three groups by 1/3 using the equal fractions method. The definitions and detailed discussion of these bridging techniques are found elsewhere [2,3].

Age, sex, region and race/ethnicity variables were used to tabulate the study population in Table 1. Age was computed using dates of birth of veterans and defined as age on January 1, 2004. The U.S. Census Bureau's definition of region and the veterans' state of residence were used to group all veterans in regions.

**Table 1:** Study Population by Patient Demographic Characteristics, All VHA Users in 2004

	N (%)		
	
	All Users	Users with SR race/ethnicity	Users with SR and OR race/ethnicity
Total	4,814,175	1,890,649	988,277

Age			
< 55	1,321,901 (27.46)	517,268 (27.36)	251,083 (25.41)
55 – 64	1,164,843 (24.20)	493,638 (26.11)	258,052 (26.11)
65 – 74	1,067,405 (22.17)	409,489 (21.66)	219,427 (22.20)
75 – 84	1,061,298 (22.05)	396,189 (20.96)	216,433 (21.90)
85 and over	198,728 (4.13)	74,065 (3.92)	43,282 (4.38)

Sex			
Male	4,425,937 (91.94)	1,785,358 (94.43)	938,471 (94.96)
Female	388,233 (8.06)	105,291 (5.57)	49,806 (5.04)
Unknown	5 (0.00)		

Region			
Northeast	768,974 (15.97)	300,152 (15.88)	156,537 (15.84)
Midwest	1,070,858 (22.24)	417,123 (21.06)	219,503 (22.21)
South	1,976,662 (41.06)	845,155 (44.70)	443,063 (44.83)
West	997,679 (20.72)	328,217 (17.36)	169,174 (17.12)
Unknown	2 (0.00)	2 (0.00)	

Race, New Standards^§^			
White	1,497,743 (31.11)	1,497,743 (79.22)	769,661 (77.88)
Black or African American	314,564 (6.53)	314,564 (16.64)	180,007 (18.21)
AIAN	7,986 (0.17)	7,986 (0.42)	3,737 (0.38)
Asian or Pacific Islander	31,310 (0.65)	31,310 (1.66)	14,262 (1.44)
Multiracial	13,135 (0.27)	13,135 (0.69)	7,347 (0.74)
By OB and PROXY	1,314 (0.03)		
Inconsistent	5,203 (0.11)		
Missing or Unknown	2,942,920 (61.13)		

Ethnicity, New Standards			
Hispanic	100,727 (2.09)	100,727 (5.33)	57,085 (5.78)
Non-Hispanic	1,743,996 (36.23)	1,577,729 (83.45)	815,150 (82.48)
Unknown	2,969,452 (61.68)	212,193 (11.22)	116,042 (11.74)

* SR indicates self-reported; OR, observer-recorded; AIAN, American Indian or Alaska Native; OB, observation; PROXY, proxy report.

^§^The percentages in the last two columns do not add up to 100% due to those who identified themselves as Hispanic but did not report a valid race value.

## Results

### Self-reported race/ethnicity data

Table 1 shows the demographic characteristics of individuals used in the analysis. There were about 4.8 million users of VHA healthcare in 2004. Valid self-reported race values were recorded for slightly less than 39% of them (1.9 million).

Of those with valid race/ethnicity data in 2004, 79.2% identified themselves as Whites and 16.6% as African Americans, which together accounted for almost 96% of all VHA users with valid race/ethnicity data. Of the remainder, 39,296 (2.1%) belonged to three other single race groups (Asian, Native Hawaiian or Other Pacific Islander, and American Indian or Alaska Native) and 13,135 (0.7%) identified themselves as belonging to two or more races. The rest (1.2%) reported ethnicity but not race.

About 5.3% of users with valid race or ethnicity values in 2004 identified themselves as Hispanic. Of these Hispanics, over 25% did not report any race value and 0.6% reported inconsistent values.

Among all VHA users in 2004, only 5,203 (0.11% of all VHA users with valid self-reported race) had inconsistent self-reported race values from record to record, indicating an extremely high internal consistency (99.9%) of self-reported race values for all individuals across different healthcare records. These multiple discrepant records were not used in any comparisons between old and new data.

We could link 988,277 individuals who had a single self-reported race value in 2004 to the healthcare records with old race values from 1997 – 2002. They represent 52% of 2004 users with valid self-reported race values and 28% of all those with valid observer-recorded values in 1997 – 2002 (N = 3,494,761). Table 1 shows the breakdown of these individuals in age, gender and region categories. Females reported race at a significantly lower rate than males (27% vs. 40%; p < 0.001). Individuals in the South reported proportionately more, and those in the West reported less, self-reported races than those in other regions. While 43% of all users in the South reported valid race, only 33% of those in the West did so. Only about 16% of all users in the West could be linked across years and had both old and new race values, whereas about 20 to 22% of all users in other regions could.

### Accuracy of observer-recorded race

Table 2 shows comparison of observer-recorded with self-reported races using old race categories. Under the old standards, race had been recorded in six race/ethnicity categories: Hispanic White, Hispanic Black, American Indian or Alaska Native (AIAN), Asian or Pacific Islander (API), Black or African American, and White. Under the new standards, race is recorded in five categories, including AIAN, Asian, Black or African American, Native Hawaiian or Other Pacific Islander, and White. As far as the race categories are concerned, the only change involved splitting the old API category into separate "Asian" and "Hawaiian or Other Pacific Islander" categories in the new standards. Thus, the new standards provide slightly finer breakdowns in race/ethnicity groups than the old.

**Table 2:** Comparison of Observer-Recorded and Self-Reported Race Values*

Observer-Recorded Race	Self-reported Race**						
	
	Hispanic White	Hispanic Black	White	AA	AIAN	API	Total
Hispanic White	**32,631**^§^	1,173	7,472	442	100	896	42,714
	**82.97%**	47.57%	1.02%	0.25%	2.89%	6.88%	
	**76.39%**	2.75%	17.49%	1.03%	0.23%	2.10%	

Hispanic Black	1,473	**657**	712	399	9	70	3,320
	3.75%	**26.64%**	0.10%	0.22%	0.26%	0.54%	
	44.37%	**19.79%**	21.45%	12.02%	0.27%	2.11%	

White	4,883	83	**712,524**	9,638	2,013	6,160	735,301
	12.42%	3.37%	**97.56%**	5.43%	58.20%	47.29%	
	0.66%	0.01%	**96.90%**	1.31%	0.27%	0.84%	

AA	203	546	8,160	**166,876**	213	1,256	177,254
	0.52%	22.14%	1.12%	**93.99%**	6.16%	9.64%	
	0.11%	0.31%	4.60%	**94.15%**	0.12%	0.71%	

AIAN	64	4	937	116	**1,100**	51	2,272
	0.16%	0.16%	0.13%	0.07%	**31.80%**	0.39%	
	2.82%	0.18%	41.24%	5.11%	**48.42%**	2.24%	

API	76	3	526	70	24	**4,592**	5,291
	0.19%	0.12%	0.07%	0.04%	0.69%	**35.26%**	
	1.44%	0.06%	9.94%	1.32%	0.45%	**86.79%**	

Total	39,330	2,466	730,331	177,541	3,459	13,025	966,152

* AA indicates African American; AIAN, American Indian or Alaska Native; API, Asian or Pacific Islander.

** The race/ethnicity categories in this table are consistent with the old VA standards used before May 2003. The race/ethnicity categories in the self-reported data were mapped to the old standards as follows: Hispanic ethnicity and White and Black races were combined respectively into Hispanic White and Hispanic Black, and Asian and Native Hawaiian or Other Pacific Islander race groups were combined into Asian and Pacific Islander. Veterans with multiracial values, veterans with race values reported by observation or proxy, veterans with missing or unknown race values, and veterans who reported Hispanic ethnicity but no or non-AA minority race are excluded.

^§ ^Each cell in this table has three numbers; the first number indicates frequency; the second number, column percent; the third number, row percent.

In Table 2, the new race and ethnicity values were combined into the old categories (e.g., Hispanic ethnicity and Black or African American race were combined into Hispanic Black) and two race categories in the new standards, Asian and Native Hawaiian or Other Pacific Islander, were combined into Asian or Pacific Islander. Since those who reported multiracial values (7,347) or Hispanic ethnicity but no race or a non-African American minority race (14,430) in the 2004 data could not be assigned to the old race/ethnicity categories, they were not included in this table.

Those in the cells on the main diagonal were the individuals whose new and old race values agreed and accounted for 95.1% of all users with both new and old race values. This indicates that observer-recorded race values have an excellent agreement with the self-reported values (kappa = 0.87). There were no differences in agreement rates between males and females, but considerable variations existed between regions (results not shown). The Midwest region had the highest agreement rate (97%; kappa = 0.89) and the West the lowest (93%; kappa = 0.85).

All off-diagonal cells indicate the number of disagreements in race coding between new and old data. The largest number of divergences occurred between Whites and African Americans, the two largest racial groups. 8,160 Whites (1.1%) were coded as African Americans and 9,638 (5.4%) African Americans as Whites; these together represent 36.2% of all miscoded values in the observer-recorded data. The second largest divergence occurred between Whites and Hispanic Whites; 7,472 Whites (1.0%) and 4,883 Hispanic Whites (12.4%) were miscoded in the old data. These together represent another 25% of all miscoded races. For AIANs and APIs, almost 70% of all observer-recorded data in these two categories were miscoded. And 58% of AIANs and 47% of APIs in particular were coded as Whites in the observer-recorded data. Of the total 47,772 individuals with incorrectly identified race/ethnicity in the observer-recorded data, 40,584 (85%) were either other race/ethnicity members incorrectly identified as Whites (22,777) or Whites incorrectly identified as other race/ethnicity members (17,807).

Table 3 shows summary measures of accuracy of the old race values compared with the self-reported race values as the gold standard for the same individuals analyzed in Table 2. The summary measures were computed separately for each race category. The largest two racial categories, White and African American, had 97.6% and 94.0% sensitivity rates, respectively. For Whites, the specificity (90.3%) was considerably lower than the sensitivity (97.6%) due to a large number of individuals who were incorrectly coded as Whites in the old data. Other measures of accuracy for these two groups were either acceptable or quite good. Kappa coefficients for Whites and African Americans were 0.89 and 0.93, respectively.

**Table 3:** Accuracy of Observer-Recorded Race Values Using Self-Reported Race Values as the Gold Standard* (N = 966,152)

Race	Observed	Self-reported**		Accuracy and Agreement Measures (95% CI)				
		
		Yes	No	Sensitivity	Specificity	PPV	NPV	Kappa
Hispanic White	Yes	32,631	10,083	83.0	98.9	76.4	99.3%	0.786
	No	6,699	916,739	(82.6 – 83.3)	(98.9 – 98.9)	(76.0 – 76.8)	(99.3 – 99.3)	(0.783 – 0.790)
Hispanic Black	Yes	657	2,663	26.6	99.7	19.8	99.8	0.225
	No	1,809	961,023	(24.9 – 28.4)	(99.7 – 99.7)	(18.4 – 21.2)	(99.8 – 99.8)	(0.210 – 0.239)
White	Yes	712,524	22,777	97.6	90.3	96.9	92.3	0.885
	No	17,807	213,044	(97.5 – 97.6)	(90.2 – 90.4)	(96.9 – 96.9)	(92.2 – 92.4)	(0.884 – 0.886)
AA	Yes	166,876	10,378	94.0	98.7	94.1	98.6	0.927
	No	10,665	778,233	(93.9 – 94.1)	(98.7 – 98.7)	(94.0 – 94.3)	(98.6 – 98.7)	(0.926 – 0.928)
AIAN	Yes	1,100	1,172	31.8	99.9	48.4	99.8	0.382
	No	2,359	961,521	(30.3 – 33.4)	(99.9 – 99.9)	(46.3 – 50.5)	(99.7 – 99.8)	(0.366 – 0.398)
API	Yes	4,592	699	35.3	99.9	86.8	99.1	0.498
	No	8,433	952,428	(34.4 – 36.1)	(99.9 – 99.9)	(85.8 – 87.7)	(99.1 – 99.1)	(0.489 – 0.506)
Hispanic, Combined	Yes	35,934	10,100	86.0	98.9	78.1	99.4	0.810
	No	5,862	914,256	(85.6 – 86.3)	(98.9 – 98.9)	(77.7 – 78.4)	(99.4 – 99.4)	(0.807 – 0.813)
AIAN & API Combined	Yes	5,767	2,146	35.0	99.8	72.9	98.9	0.467
	No	10,717	968,132	(34.3 – 35.7)	(99.8 – 99.8)	(71.9 – 73.9)	(98.9 – 98.9)	(0.459 – 0.475)
Non-White, non-AA Combined	Yes	42,923	10,674	73.7	98.8	80.1	98.3	0.753
	No	15,357	897,198	(73.3 – 74.0)	(98.8 – 98.9)	(79.7 – 80.4)	(98.3 – 98.3)	(0.750 – 0.756)

* CI indicates confidence interval; PPV, positive predictive value; NPV, negative predictive value; AA, African American; AIAN, American Indian or Alaska Native; API, Asian or Pacific Islander. Veterans with multiracial values, veterans who reported Hispanic ethnicity but no or non-AA minority race, veterans with race values reported by observation or proxy after May 2003 and veterans with missing or unknown race values were excluded.

** In self-reported data, Hispanic ethnicity and White or Black race were used to identify respectively Hispanic White or Hispanic Black in this table. Both Asian and Native Hawaiian or Other Pacific Islander in the self-reported data are used to identify Asian or Pacific Islander.

Sensitivity rates for other race/ethnicity groups were considerably lower. Hispanic Whites had 83% sensitivity, but other groups had sensitivities lower than 40% (AIAN, 31.8%; API, 35.3%; Hispanic Black, 26.6%), indicating that they are not reliable enough for research use as separate race/ethnicity groups.

Race categories are frequently combined into larger ones in research. The last three rows in Table 3 shows the accuracy and agreement measures for combined race/ethnicity categories. The sensitivity for the Hispanic category that combines Hispanic White and Hispanic Black was 86% (kappa = 0.81). The sensitivity for the combined AIAN and API category was still extremely poor at 35% (kappa = 0.47). All non-African American minority groups combined into one category were only 73.7% sensitivity (kappa = 0.75). When Hispanics with no race, multiple races, or non-African American minority races were included in these comparisons, the summary measures showed slightly worse agreement between the old and new data with sensitivity rates 85% (kappa = 0.83) and 70% (kappa = 0.74) for the combined Hispanic category and the combined non-White, non-African American category, respectively (data now shown).

Table 4 shows the extent of agreement between the self-reported and observer-recorded race/ethnicity values in various race/ethnicity breakdowns. When the groups are divided into White, African American, Hispanic, and Other, the two agreed for 95.2% of all individuals (kappa = 0.88). This did not improve much in other groupings such as White, African American, and Other (95.3%; kappa = 0.88) and White and Other (95.7%; kappa = 0.89). African American and non-African American classification had an agreement of 97.8% (kappa = 0.93), by far the highest among all combinations considered.

**Table 4:** Overall Agreement of Observer-Recorded Race Values with Self-reported Race in Various Groupings* (N** = **979,415)

Race/Ethnicity Grouping	Number Agreeing	Percent Agreeing	Kappa
White, AA, Hispanic, Other	932,315	95.2	0.881 (0.880–0.882)
White, AA, Other	933,652	95.3	0.884 (0.883–0.885)
White, Other	937,129	95.7	0.885 (0.884–0.886)
AA, Other	958,140	97.8	0.927 (0.926–0.929)

*AA indicates African American. Veterans with multiracial values and veterans with Hispanic ethnicity and non-AA minority races were excluded.

### "Bridging" issues

Another important issue in the transition to the new standards is how to use the multiracial values in tabulating trends for multiple years. We considered four commonly used bridging methods to assign multiracial individuals to single race categories in the old standards. No matter what method was used, these assignments had little effect for the two largest race groups, Whites and African Americans. However, when the subjects categorized as multiracial were allocated to the smallest group, the number of individuals in the AIAN group increased by almost 55% and the API group by 20% (Table 5). Some other methods also increased these two groups by 10% to 29%, indicating that the allocation methods can potentially have a large impact in race/ethnicity identification for API and AIAN groups when individuals with multiple races in the self-reported data are "bridged" to the single races in the old, observer-recorded data.

**Table 5:** Racial Distribution of Veterans Before and After Allocation of Multiracial Individuals by Using 4 Bridging Techniques

	Number (%) Before "Bridging"^a^	Number (%) After "Bridging" Using 4 Techniques			
		
		Smallest Group^b^	Largest Group Other Than White^c^	Largest Group^d^	Fractional Assignment – Equal Fractions^e^
White	1,431,498 (80.53)	1,431,498 (79.94)	1,431,498 (79.94)	1,442,992 (80.58)	1,437,203 (80.25)
AA	310,288 (17.45)	314,006 (17.53)	315,453 (17.62)	311,547 (17.40)	312,838 (17.47)
AIAN*	7,173 (0.40)	11,093 (0.62)	10,440 (0.58)	7,173 (0.40)	9,100 (0.51)
API*	28,707 (1.61)	34,204 (1.91)	33,410 (1.87)	29,089 (1.62)	31,661 (1.77)

Total	1,777,666 (100.00)	1,790,801 (100.00)	1,790,801 (100.00)	1,790,801 (100.00)	1,790,801 (100.00)

* AA indicates African American; AIAN, American Indian or Alaska Native; API, Asian or Pacific Islander.

^a ^Veterans with multiracial values and Hispanic ethnicity excluded.

^b ^Assigns multiracial values to the smallest group.

^c ^Assigns multiracial values to the largest group other than White.

^d ^Assigns multiracial values to the largest group.

^e ^Assigns equal weights to each racial group identified. Does not add up to total due to rounding errors.

## Discussion

This study examined issues related to the transition to the new federal standards in collecting race/ethnicity in the VA. We showed that the *overall *agreement between the observer-recorded and self-reported race/ethnicity data was excellent. Excluding those who reported ethnicity only in 2004, the overall agreement between the new and old data was over 95% (kappa = 0.87). This indicates that the observer-recorded data are highly consistent with, and can be used together with, the self-reported data without creating substantial bias in multi-year trends. This was mainly due to accurate identification by observation of the two largest racial groups, Whites and African Americans, who had sensitivity rates of 97.6% (kappa = 0.89) and 94.0% (kappa = 0.93), respectively.

However, we also showed that observation was not a reliable method of identifying race/ethnicity for non-African American minority groups. The sensitivity rates for these groups varied between 26.6% and 83.0% (kappa, 0.23 and 0.79), too low for identifying them separately for research purposes. They can be combined with other groups to create a higher-level, more inclusive group, to achieve better sensitivity. We showed that the African American and Other (Whites and all other non-African American minorities combined in one group) distinction had the best agreement between the old and new race/ethnicity data.

We also observed a systematic pattern by which observer-recorded data misclassified individuals; 85% of all inaccurate race/ethnicity in the observer-recorded data involved Whites in such a way that Whites were incorrectly identified as members of a minority group or vice versa. This pattern of misclassifications in the observer-recorded data can reduce the observed disparity between Whites and other racial groups, and accordingly the racial disparity based on observer-recorded data may be underestimated. Researchers using the observer-recorded and self-reported data together thus need to conduct sensitivity analyses to rule out the possibility that any change in disparity before and after the transition is not attributable to using mixed data.

The findings of this study are consistent with a previous study that reported agreement rates of 97.9% and 92.0% for Whites and African Americans, respectively, between the observer-recorded race in VA administrative files and the self-reported race in a survey of veterans [4]. The agreement of the APIs was much lower with the self-reported data in the administrative files (35.3%) than with the survey data (75.5% for Asians and 69.6% for Pacific Islanders).

The observer-recorded race/ethnicity data in the VA Medical SAS Datasets also compare favorably in accuracy with those in the Medicare Enrollment Database (EDB), which showed 96.5% and 95.6% for sensitivity rates for Whites and African Americans, respectively [22]. The VA observer-recorded data performed slightly better in identifying Whites but slightly worse in identifying African Americans. In the VA, only about 15% of Hispanics were misclassified to some other race/ethnicity groups, while almost 65% in the EDB were misclassified. The sensitivity for Hispanic category in the EDB was only 35.7% compared with 85.5% in the VA data. Except for Asians, the sensitivity rates for other minority groups in the VA data were much higher than those in the EDB. Thus, when both VA and Medicare race values are available for an individual, this implies that the old VA data should actually be preferred to the Medicare data, especially for Hispanics.

We found that the completeness of self-reported race/ethnicity data was a serious problem. Over 60% of all VHA users in 2004 did not report any race values, which represents almost 15% drop in completeness compared with observer-recorded data in the pre-transition years. For example, 45% of all VHA users in 2002 were missing race/ethnicity data. This sudden drop in completeness of the race data from the pre-transition years may in part be a transitional problem that occurs during the first few years after a new system is implemented. If these were the case, the race/ethnicity data may be randomly missing.

However, it is also possible that some groups may not like to disclose their race/ethnicity more than others and so this drop may also be in part attributable to the change in data collection methods. As we have shown, race/ethnicity data for multiracial individuals may be seriously underreported in the VHA data. Only 0.3% of all users and 0.7% of those with valid self-reported race/ethnicity values reported two or more races in the 2004 VHA data, while a national survey of veterans conducted in 2001 indicated that 2.1% of all veterans and 3.2% of VHA users may be multiracial [23]. The selective self-reporting is also shown in the regional variations in the completeness of race data in 2004. The South region had the highest completeness at 43%, followed by Northeast and Midwest at 39%, and West at 33%. According to the 2000 Census, the West had the highest concentration of multiracial persons with 40% of all multiracials in the country [24]. This suggests that the multiracial individuals are more reluctant to report their own races than individuals of single race and the self-reported data may have selection issues that the previous observer-recorded data do not have, further complicating the mixed use of observer-recorded and self-reported data for multi-year trends.

To address the incompleteness issue, the VA can consider several options. First, the VA can obtain data through special surveys or from external sources. As the Centers for Medicare and Medicaid Services (CMS) have done [25,26], the VA could survey veterans specifically to collect race/ethnicity data from enrollees whose self-reported race/ethnicity data are not known. Alternatively, the VA could establish an interagency agreement with the Social Security Administration (SSA) and acquire the SSA's race data regularly to supplement its own race data, an approach also used by the CMS [22,26].

However, a more fundamental and long-term solution to this problem is to improve race reporting at the source, namely, in VA hospitals and clinics. The VA may need to examine whether the way the race/ethnicity questions are asked (e.g., specific wording of the questions, use of any prefatory remarks or probes following an incomplete answer, or circumstances under which the questions are asked) can be improved. Previous research suggests that how a question is asked about race/ethnicity can make substantial differences in the response rate, especially for small race/ethnicity groups [27,28]. For example, a study showed that an open-ended question (i.e., allowing the respondents to describe their race/ethnicity in their own terms) can reduce the rates of unusable data compared with data obtained with the OMB standards, and that the open-ended format is especially effective in improving race reporting for minority groups such as Hispanics, Asians or multiracial individuals, who are often reluctant to describe their race/ethnicity profile in pre-defined categories such as those in the OMB standards [27].

Until the self-reported race data in the VA are substantially improved in completeness, researchers using the VA race data may consider supplementing self-reported data with observer-recorded data from past years or through SSA or Medicare data sets when applicable. Future research needs to examine how the observer-recorded and self-reported data can be integrated for a well-validated patient-level race database and if such an approach can substantially improve the completeness of VA race data.

In the meantime, however, whether self-reported data are used alone or in combination with old race/ethnicity data, users of VA self-reported race data should be aware of the potential selectivity in the self-reported race/ethnicity data. As discussed above, about 25% of those who reported Hispanic ethnicity did not report race in the FY2004 VHA data. These individuals may not view themselves as having racial identity distinct from their ethnicity [27]. They thus may choose either "Other" category for their race or refuse to disclose race when they are given OMB categories. This is shown in 2000 Census data in which over 42% of all those who reported their ethnicity chose "Some other race" compared with only 5.3% of the total population [29]. In the VHA, "Other race" is not provided as a response category and as a result many refused to report race. Regional variations in the completeness of self-reported race/ethnicity data (e.g., 33% in the West vs. 41% in the other three regions) may also reflect not so much systemic failure to enforce the new race/ethnicity data collection standards among VHA facilities in the West as variations in the distribution across regions of non-African American minority groups such as Hispanics, Asians, and individuals of two or more races.

One limitation of this study is that we have not considered the characteristics of the VA population who had no self-reported data. Their individual characteristics, and accordingly the accuracy of their observer-recorded race values, may be systematically different from those who could be linked. As a consequence, this study cannot provide an estimation of how good the quality of data would be that combine the old and new information. Further, the findings about the accuracy of observer-recorded race need to be cautiously generalized because only about 28% of all valid observer-recorded data for 1997 – 2002 could be linked to the self-reported data.

## Conclusion

In conclusion, our results indicate that observer-recorded race data for VA users compiled from 1997 – 2002 have excellent agreement with self-reported race data collected in 2004 for veterans of African American or White race. However, there is considerable under-reporting of race overall and the accuracy of observer-recorded race data for non-African American minorities is poor, limiting the usefulness of observer-recorded race data for individuals in these racial categories. Private and public healthcare providers considering a similar transition can learn from the VA experience in anticipating potential issues and planning for a smooth transition.

## Abbreviations

AIAN – American Indian or Alaska Native

API – Asian or Pacific Islander

CMS – Centers for Medicare and Medicaid Services

EDB – Medicare Enrollment Database

OMB – Office of Management and Budget

SSN – Social Security Number

VA – Department of Veterans Affairs

VHA – Veterans Health Administration

## Competing interests

The author(s) declare that they have no competing interests.

## Authors' contributions

MWS participated in the design of the study, oversaw statistical analyses of data, and drafted the manuscript.

HZ was responsible for data collection, management, statistical analysis, and edited the manuscript.

NA participated in the design of the study, data analysis, and edited the manuscript.

KS participated in the design of the study, the interpretation of results, and edited the manuscript.

BCT participated in the design of the study and edited the manuscript.

TJW oversaw the 1^st ^grant (NIDDK), participated in the design of the study, the interpretation of results, and edited the manuscript.

DMH oversaw the 2^nd ^grant (SDR 98-004), participated in the design of the study and the interpretation of results, and edited the manuscript.
